# Exploring the relationship between entertainment and education in anatomy public engagement: A qualitative examination of anatomists' perspectives

**DOI:** 10.1002/ase.70092

**Published:** 2025-07-09

**Authors:** Lucas D. Wilmshurst, Lauren Clunie, Kieron Brand, Chandini Parsan Chand, Kat A. Sanders

**Affiliations:** ^1^ Centre for Anatomical and Human Sciences Hull York Medical School, University of Hull Kingston upon Hull UK; ^2^ NHS Grampian Health Board Aberdeen UK; ^3^ Northumbria NHS Foundation Trust Newcastle upon Tyne UK; ^4^ Leeds Teaching Hospitals NHS Trust Leeds UK; ^5^ School of Medical Sciences, Faculty of Medicine & Health University of Sydney Camperdown New South Wales Australia

**Keywords:** anatomy, education, edutainment, entertainment, public engagement

## Abstract

Entertainment is deeply rooted in education, from wise‐cracking teachers to health documentaries. In the context of anatomy, this already complex relationship is entwined with deeply significant ethical considerations, often related to the field's reliance on human tissue, yet it remains unexplored. This study aimed to understand anatomists' perceptions of the role of entertainment in anatomy public engagement. Semi‐structured interviews were conducted with seven anatomists with strong public engagement profiles, and thematic analysis was conducted on the transcripts. There was variability between participants as to what “entertainment” means and its link to education, though a range of ways educators can be entertaining, including narratives, personal relevance, and games, were identified. There were also two perceived impacts of entertainment on audiences identified: impact on education (to aid or impede learning) and impact on engagement including initial engagement, engagement throughout, and ongoing engagement. This study found that anatomists are keenly aware of the historical and anatomical context of their public engagement, with some hesitancy to even use the term “entertainment” due to misconceptions that it is inherently hedonistic and thus disrespectful. This study highlights the complex and context‐dependent nature of the relationship between entertainment and education in anatomy public engagement, emphasizing the need for anatomists to carefully consider the types and impacts of entertainment they employ to effectively engage and educate public audiences while respecting the discipline's unique ethical considerations. More research is needed to clarify this relationship between education and entertainment from the audience's perspective.

## INTRODUCTION

Teaching is a performance, so they say, and entertainment is beneficial in educational settings, having been shown to increase both motivation to learn and retention of information.^1^, ^2^, ^3^ Entertainment in education has been explored in numerous formal education contexts^4^, ^5^ and includes exploring the use of humor to aid knowledge retention,^4^ medical quizzes^6^, ^7^ and anatomy board games,^8^ all of which have been shown to successfully promote learning.^6^, ^7^, ^9^ However, education to the wider public plays by slightly different rules.

Entertainment‐Education is the use of entertaining media such as films, television shows, and radio programs to educate the public. These media primarily aim to entertain but include educational content,^10^ and as the focus is not primarily to learn, it results in a different type of learning compared to formal education. For example, nudging audiences toward safer sexual practices or engagement with health screening.^11^ Conversely, education for the public can also be delivered in such a way that the educational message is foregrounded rather than the entertainment; this is science communication. Diverse examples include documentaries, science journalism, and TED talks. Public engagement, a subset of science communication, prioritizes dialogue and audience involvement over the predominantly one‐way communication seen in televised documentaries and written materials. Public engagement emphasizes the *engagement* aspect; the focus is on the importance of dialogue and involving the audience in two‐way conversations.^12^

Within anatomy education, public engagement presents unique concerns, owing to its reliance on human tissues and the field's long and complex history. The historical connection between anatomical education and the public is a troublesome one. In the 14th century, anatomical dissection was legalized by the Roman Empire and bodies were sourced from condemned criminals for medical students.^13^ However, by the late 15th century, these dissections evolved into extravagant events, where anyone who could afford to go would gather for a public execution, then watch the body be dissected, finishing with a banquet.^14^ As dissections became more popular, the pressure on acquiring bodies for dissection drastically increased, which contributed to the rise of more sinister means, including grave robbing^14^ and murder. The Anatomy Act of 1832, prompted by the murders committed by Burke and Hare in Edinburgh, permitted the use of unclaimed bodies in the United Kingdom.^15^ However, while this eased the burden on medical schools, it resulted in the bodies of those who lived in poverty, worked in workhouses, or died in slavery being used without consent.^14^ While large swathes of the Western world have since moved to body donation practices grounded in informed consent,^16^ the perception of anatomy as inherently gruesome and sordid remains in the public subconscious.

Much of the anatomical information public audiences receive comes from news media, where anatomical stories are often sensationalized. The framing of news stories shapes public perceptions,^17^ and anatomy often suffers from portrayals of gruesomeness and morbid curiosity linked to scandals and historical injustices.^18^ However, news articles featuring contributions from anatomists tend to offer more balanced presentations.^19^ Therefore, public engagement in anatomy is vital to rectify misconceptions about the discipline, and anatomists are best placed to deliver such events to ensure the knowledge received is accurate. Good anatomical knowledge among the public can boost health literacy and strengthen relationships with health care practitioners.^12^ However, anatomical public engagement has a big task ahead of it, as studies have found that public knowledge of basic anatomy (i.e. the position of organs) is limited.^20^, ^21^

A notable example of anatomy public engagement is Gunther von Hagens' Body Worlds. This museum‐like exhibition showcases plastinated human specimens to educate the public about the wonders of human anatomy^22^, ^23^ and aims to democratize anatomy education by making examples of human anatomical dissections accessible to all.^24^ However, the use of human tissue, especially in the context of the discipline's history, is the focus of intense debate. Some anatomists argue von Hagens' defense,^25^, ^26^, ^27^ but a majority argue that Body Worlds sensationalizes and trivializes the subject.^22^, ^28^, ^29^ In addition to Body Worlds, Gunther von Hagens performed the first public dissection in 170 years and the first televised dissection in 2002. This sparked ethical debates about anatomical dissection and sensationalism.^30^ The 2022 airing of the My Dead Body documentary by Smith et al.^31^ prompted further discussion within the anatomy community about the ethics of public dissection^18^ and the complex path our field faces to find an ethical framework for public dissections.^32^

Body Worlds and public dissection of human tissue, however, does not hold a monopoly on anatomy public engagement; other outreach activities are flourishing.^33^ There is a vast array of public engagement formats, including free online courses,^34^ in‐person events^35^, ^36^ and a range of podcasts, books, and videos. There are also many potential resources that anatomy public engagement can use, including: arts and crafts,^37^, ^38^ interactive software, dissection of animal tissues,^35^ games,^39^ and even making mini skeletons out of pasta shapes.^12^

To summarize, there is a clear need for anatomical public engagement that aims to educate the public about their own bodies, correct misconceptions, and start a dialogue with the public, and anatomists are meeting this demand with increasingly inventive and interactive methods. However, despite its educational benefits, the role of “entertainment” in these activities and the cultural position of anatomy public engagement as a source of entertainment is absent in the literature.

This study intends to fill this research gap by examining the perspectives of anatomists involved in public engagement and their attitudes toward the use of entertainment. It aims to answer the following research questions: *what are anatomy educators' views toward the use of entertainment in public engagement? What role does entertainment play in anatomy public engagement?*

## METHODS

The study's aims were achieved by recruiting a sample of anatomists heavily involved in public engagement, conducting a series of interviews with them, and thematically analyzing these discussions.

### Research paradigm

A research paradigm explains how a researcher understands what is knowable, from positivism assuming there is an objective reality that can be measured, through to interpretivism assuming that each individual has their own subjective reality. The research paradigm underpins their methodologies as they develop new knowledge; positivist research uses quantitative methods and aims to eliminate bias, whereas interpretivist research uses qualitative methods and acknowledges that bias is unavoidable.^40^

This article uses a soft social constructionist research paradigm (a paradigm close to the interpretivism end of the spectrum). Social constructionism assumes that realities (and thus our knowledge about them) are constructed socially through discourse between individuals.^41^ This is the most appropriate paradigm for this article's research question, as “entertainment” is a socially constructed concept. Ask people what kind of music or comedy they enjoy, and it quickly becomes clear that the experience of entertainment is incredibly subjective and personal. Social constructionism places emphasis on the quality and meaning of data, rather than its quantity or statistical validity. By its very nature, social constructionism acknowledges that participants' and researchers' backgrounds shape their outlooks and seeks to interrogate this rather than eliminate bias.

This research paradigm has guided the use of qualitative research methods, seeking not to discover absolute truths about the role of entertainment in anatomy public engagement, but to collect and analyze a variety of subjective accounts to develop a picture of how entertainment is perceived and understood by those with personal experience. It has also guided the use of thematic analysis to explore which concepts recur between different participants to discover areas of agreement and disagreement.

### Participant recruitment

Participants were selected via purposive sampling to maximize insight into the research topic.^42^ To improve research credibility, maximal variation sampling was employed to recruit a diverse sample of knowledgeable individuals representing various academic and public engagement backgrounds. In alignment with the research paradigm, depth of experience was prioritized over quantity of participants, and participants were selected based on their extensive public engagement experience across various formats, including writing, media presenting, live events, and other novel forms of activities. Further to this, some participants were selected for their extensive knowledge and prior research on anatomy public engagement, as their depth of knowledge could inform discussions of the theoretical relationship between entertainment and education. Participants were identified from their public profiles of anatomical public engagement work. While this convenience sampling is not entirely purposeful, only those meeting the criteria and presumed to have distinctive or detailed views on public engagement were invited for interviews.

The Hull York Medical School Ethics Committee (committee reference 21 27) granted ethical approval for the study. Verbal and written consent were obtained before each interview. As some participants took part in recognizable projects, this report was carefully written to exclude identifiable information.

### Data collection

One‐to‐one semi‐structured interviews were employed to enable in‐depth exploration with participants while ensuring that key topics were still covered.^43^ An interview protocol (Appendix A) was developed using findings from the literature review and included several key areas to be explored with each participant.^44^

Interviews were hosted and recorded online using virtual platforms Microsoft Teams and Zoom to facilitate the inclusion of anatomists outside the authors' geographical location. To enhance research dependability, the recordings were transcribed by the lead researcher (LDW) and sent to each participant to confirm accuracy.

### Data analysis

The interview transcripts were analyzed using Braun and Clarke's^45^ framework of thematic analysis. Initial semantic and latent codes were produced from each transcript by the lead researcher (LDW), with similar ideas identified early and interlinking codes collated. Coded data extracts were grouped together to form potential themes. Anonymized transcripts were shared with fellow investigators (KB and CPC), who independently coded them, enabling second‐rater discussion and refinement of the initial coding. Initial themes were reviewed by the whole research team: first at the level of the coded data, ensuring extracted data comfortably fit into the themes, and then at the dataset level to ensure accurate representation. Clear definitions and names of themes were then developed to concisely represent the findings.

This use of thematic analysis relies heavily on reflexivity and openness.^46^ More positivist methods of thematic analysis assume that themes lie dormant in the raw data, waiting for an eager researcher to coax them out. Reflexive thematic analysis, the method preferred for this study, recognizes that themes are developed from the active engagement of a researcher with their data. Reflexive practice is thus a crucial element throughout this process, so a reflexive journal was kept throughout to chart the lead researcher's experience with the dataset. Early ideas for semantic themes were noted down during the transcription process, and ideas were revisited as latent themes were developed. A reflexivity statement in first‐person is provided below to situate this research within the backgrounds of the lead researcher (LDW) and the wider research team.

### Reflexivity statements

As an anatomy student with an interest in education, my background may have influenced the course of this research. My personal interest in education meant my focus was predominantly on how educators perceive entertainment. This informed this project's focus on educators' perceptions, but ultimately limited the scope of the project where it may have benefited from considering the audience's perspectives as well.

I have a strong interest in public engagement, particularly in written science journalism. Thus, my research may have over‐emphasized the role of written forms of public engagement. This underpinned an active choice to seek as wide a group of participants as possible, including those whose focus is primarily on teaching live public audiences and those who focus on art‐based engagement. Researchers LDW, KB, and CPC were all in education at the time of conducting this research. However, like KAS and LC, we were actively involved with educating medical students and were consequently predisposed to thinking about entertaining education from the perspective of the educator.

All participants and researchers are based in Western nations, live relatively comfortable lives, and to the best of our knowledge, are able‐bodied. This research, therefore, could not explore avenues such as how attitudes toward entertainment in public engagement may vary in developing nations, or how some types of entertainment may be inaccessible to some educators or audiences. This does not diminish the richness and quality of this article per se, but is an area that warrants exploration in further research.

Prior to this project, I had never conducted interviews before. I therefore conducted a mock interview with my primary supervisor (KAS) to improve my interviewing skills and ensure I was getting the most out of this primary data collection. These interviews were with several eminent anatomists, including people I have great personal respect for. This could have resulted in a power imbalance, where I may have shied away from asking more challenging questions.

This was also my first time conducting thematic analysis. Coming from a medical background, it challenged the positivist assumptions I had previously operated in. Thematic analysis proved to be more challenging and rigorous than I anticipated. Despite being new to qualitative research, I read the underlying theory of thematic analysis and partook in training in interview techniques and thematic analysis to gain these essential research skills.

## RESULTS

### Participants

Of 10 anatomists invited, seven were interviewed. P1, P2, P3, P5, and P7 were UK‐based, while P4 and P6 resided in Australia and the USA, respectively. All seven participants had strong profiles demonstrating extensive experience with various forms of public engagement in anatomy. P1 and P3 were heavily involved in televised and written formats, P2 and P7 conducted art‐related engagement, and P4 and P6 primarily hosted podcasts (see Table 1). P1 was most active in public engagement, while P5 considered it a minor aspect of their career.

**TABLE 1:** Types of engagement conducted by participants.

Participant	Types of public engagement
P1	Television, books, public lectures, community talks, science festivals, outreach
P2	Public talks, blog, museum events
P3	Television, radio, books, public lectures, legal communication
P4	Podcast, website, radio, museum events, outreach
P5	YouTube channel
P6	Science festivals, museum events, public lectures, podcasts, outreach
P7	Public talks, science festivals, museum events, outreach

### Themes

**FIGURE 1:**
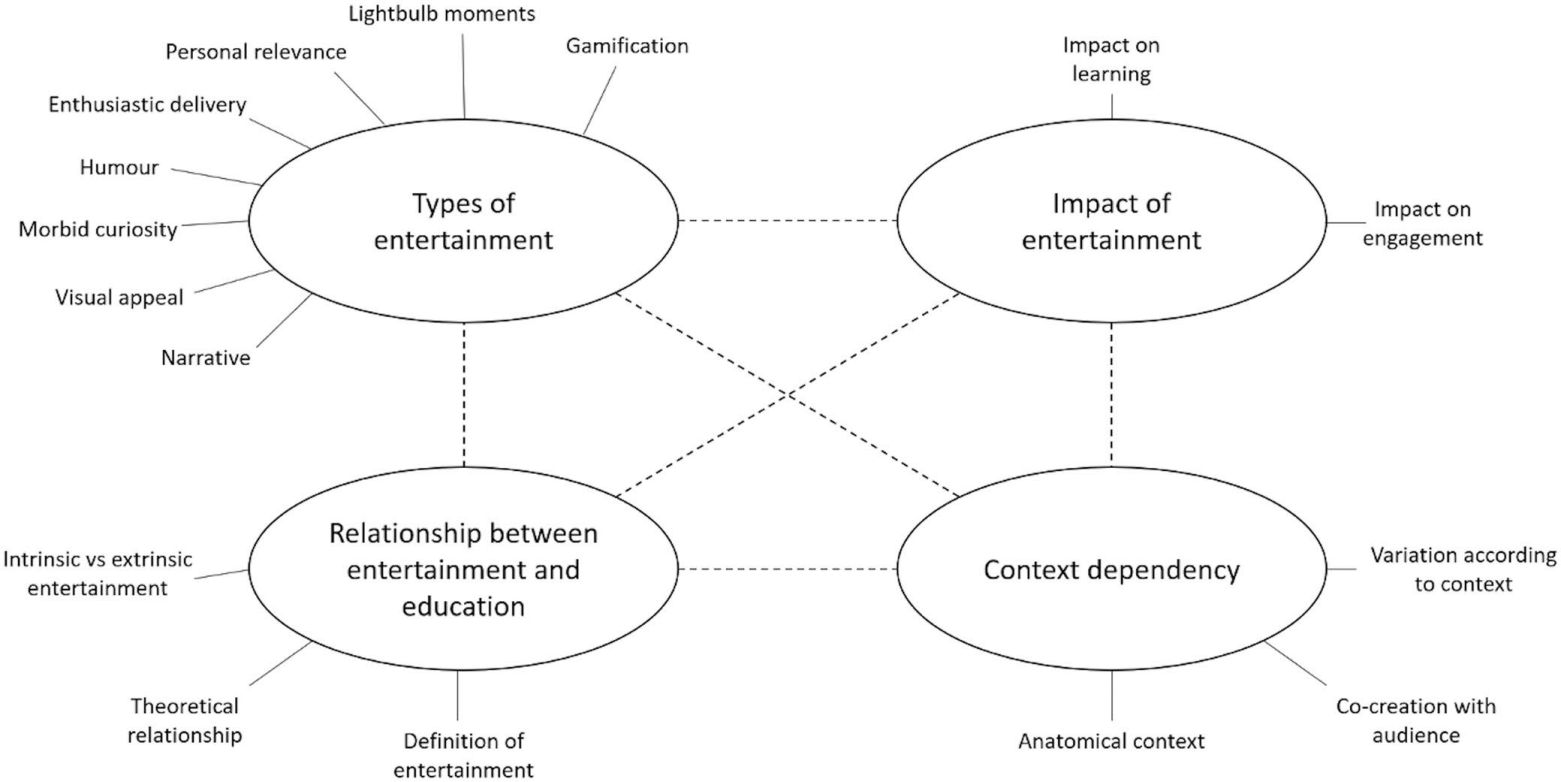
Outline of themes and subthemes.

Four main themes were developed (Figure 1), each encompassing an array of subthemes. The first theme was the *Relationship between entertainment and education*. The second was the *Types of entertainment* used in the context of anatomy public engagement. The third theme was the perceived *Impact of entertainment* on the audience. The final theme was *Context dependency*, how all of this is variable depending on the context, including the specific context of anatomy public engagement.

#### Theme 1—Relationship between entertainment and education

The intricate relationship between entertainment and education was identified as an important theme underlying much of the discussion. This theme incorporates three disparate ideas related to how entertainment is tied to education: (1) Definition of entertainment: how entertainment in this context is defined; (2) Theoretical relationship: recognition of the complex spectrum on which entertainment and education lie; and (3) Intrinsic and extrinsic entertainment: the intrinsic or extrinsic nature of entertainment relative to the educational content—whether entertaining elements are inherently part of the educational content or added on top by educators.

##### Definition of entertainment

Although numerous different definitions of “entertainment” arose, the main recurring idea was that entertainment is a communication style used by educators to engage an audience and deliver educational content.

Education is really about learning outcomes, what content needs to be addressed. And entertainment is the engagement part of that. ‘How am I going to achieve those learning outcomes?’, not ‘what are they?’ (P6)

One participant's focus on performance reiterated the idea that entertainment is about how you communicate: “*Entertainment, I guess, is just showmanship, isn't it? It's a performance… people want enjoyment from entertainment*.” (P7) Other participants discussed entertainment as synonymous with “fun” (P3) or “recreation” (P5). Only one participant discussed entertainment beyond enjoyment alone. They perceived entertainment as a form of escapism. For P2, entertainment was less grounded in reality and transported the audience to other worlds.

if you go and watch Macbeth, the Shakespeare play, that doesn't make you laugh around and roll in the aisles, but it's arguably still entertainment. But I wonder whether there's an element of because it's taking you to a different reality… that you have to be taken out of your current settings for it to classify as entertainment. (P2)

Most participants focused on entertainment as an action of the presenter. P6 discussed entertainment as being an audience experience: “*There are two ways to assess entertainment in this case. How entertaining does the performer feel they're being, versus how entertaining was the performer to the audience*.”

This was remarked on in the reflexive journal as something that had not come up in previous discussions: whether entertainment is an action or a response, a style or an emotion. Later, during development of themes, it was identified as latent in several other participants' comments, though it had not been discussed overtly.

A division arose between participants who were happy discussing their engagement as “entertainment,” and those who did not approve of the term in relation to *anatomy* public engagement:

I have a little bit of a problem with the word ‘entertainment’. Entertainment for me always sounds so trite. It sounds like a frippery. It sounds to me almost disrespectful. (P3)

Similar views were expressed by P2 and P5, who were uncomfortable describing public engagement as “entertainment” as it suggested enjoyment was the primary motive of such events: “*Maybe the difference is the presenters are entertaining as opposed to it being entertainment*.” (P2). For these participants, there was a distinction between entertainment as the primary intention of public engagement, and entertainment as a by‐product of education.

##### Theoretical relationship

There was widespread recognition of the closeness of the relationship between entertainment and education: “*I don't think they're necessarily at opposite ends of the spectrum, I think they're complementary*,” (P3). However, this relationship was recognized as a complex one that is difficult to pin down and describe: “*It's a very fine line [between entertainment and education], isn't it? And it wavers in and out*.” (P2).

There were almost as many conceptualizations of the relationship between entertainment and education as there were participants. Some conceived of the relationship as one in which entertainment is a vehicle used to deliver education to audiences:

People want enjoyment from entertainment. They're not necessarily looking to learn… you can achieve education through entertainment by stealth. (P7)

Others perceived entertainment as always being secondary to the educational content:

I think [entertainment] is more important than people give it credit for, but it's obviously still secondary to the main reason we're in this job, which is to educate. (P5)

Ultimately, the main recurring theme was uncertainty: the participants each had unclear conceptualizations of the relationship that seldom agreed with each other. The lead researcher's reflexive journal noted early in the interview process that this complexity was already a recurring theme across the initial interviews. It was notable that even in the early interviews, this was highlighted as a point of contention; not only between different participants, but even individual participants lacking clarity.

One quote that best encapsulated this complexity came from P2:

I think they're probably two different spectrums. Maybe all spectrums of one big sphere of spectrums that all work in different ways. Because I think you can be very entertaining and not at all entertaining, and you can have either of those things and neither of them be educational. And you can be educational and you can be not educational at all, but it [also] be possible to be not educational and not entertaining. So it's not two linear lines where you move along. You can go to any four points. Could be really educational and really entertaining, not educational and not entertaining, entertaining but not educational. (P2)

**FIGURE 2:**
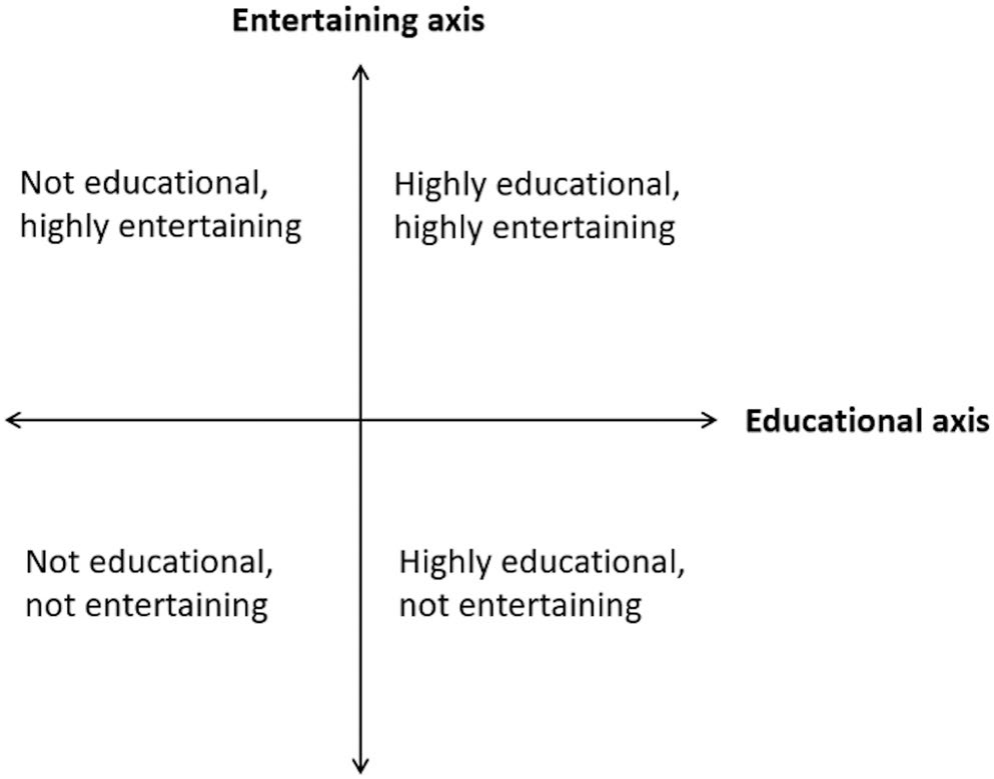
Education‐entertainment compass; a potential framework. Anatomical public engagement should fall somewhere within the top right quadrant.

This quote proposes a compass‐like two‐dimensional spectrum, with an “educational” axis and an “entertaining” axis as illustrated in Figure 2. This is supported by a quote from P6 which exemplifies that entertainment does not come solely to the detriment of education:

I will dial back learning outcomes, or dial them up, depending on who the audience is or what I'm trying to achieve…. the more entertaining I am, maybe it's because I don't have as quite as many learning outcomes that I need to achieve. Or maybe it's that I have a lot of learning outcomes that I need to achieve, and the most effective way I have found to achieve those is actually being more engaging. (P6)

These two quotes highlight how educators can position themselves at different points in the spectra, in different contexts.

##### Intrinsic and extrinsic entertainment

Several participants noted a distinction between entertainment that arose organically from within the educational content and that which was artificially added. This distinction can be described as intrinsic versus extrinsic entertainment.

Intrinsic entertainment, such as humor, playfulness, and narrative, comes from the content of the session, offering engaging perspectives on the material. Conversely, extrinsic entertainment is supplementary, such as adding *“unnecessary explosions”* (P1) to provide excitement. An example of intrinsic entertainment used by two participants was the story of the foramen ovale. Using the clinical case of the “hole in the heart” that individuals may have heard of and telling it as an interesting, memorable narrative. On the other hand, extrinsic entertainment was believed to distract from learning. For example, littering the screen with amusing but irrelevant visuals, on the other hand, would be extrinsic, as it does not help the audience understand the content at hand.

It's like taking really good ice cream and pouring on top all this junk so that you can't taste ice cream anymore. What's the point? You don't get the real value of the original ice cream, so the stick on top has no value. It might look pretty, and it might taste really good, but it doesn't add to the flavor of ice cream. (P4)

In this analogy, a high‐quality ice cream is the content and intrinsic entertainment aligns with it. This is distinguished from extrinsic “*bells and whistles*” (P1) such as visual effects and flamboyant presentation styles; the junk on top. The lead researcher's reflexive journal noted that categorization of entertainment as being intrinsic or extrinsic to the content was identified early in the transcription process as a semantic idea expressed by participants when describing ways of being entertaining.

#### Theme 2—Types of entertainment

Participants described myriad ways of making their public engagement entertaining. Each type was described as being entertaining in its own unique way and was often perceived to impact the audience uniquely too. This theme is formed from eight subthemes: narrative, visual appeal, morbid curiosity, humor, enthusiastic delivery, personal relevance, lightbulb moments, and gamification.

##### Narrative

A theme described by almost all participants was the importance of finding a narrative within the content being delivered. Narratives were perceived as entertaining because they carry audiences from one point to the next, with recognizable story beats and a flow that maintains attention.

It has to flow. You can't just have standalone segments… So rather than just a series of bullet points, you've got a piece of narrative which flows through. One idea leads to the next. (P1)

Two participants discussed how the enjoyment of narratives has an evolutionary background, with humans having evolved to recognize and value storytelling. Consequently, embedding content within a story helps make learning enjoyable, as people instinctively identify the structure and progression of a narrative.

One of the things that makes us uniquely human is the fact that we have brains that are evolved to understand storytelling… our brains connect with story better than any other form of narrative, and so, to me, engagement really means storytelling. (P6)

##### Visual appeal

Anatomy was seen by the participants as an inherently visual subject and an element of novelty was perceived to underlie the visual appeal. “*Even if you don't know the anatomy, there's something quite mesmerizing about it*.” (P7). Indeed, there was a belief that anatomy engagement can be fun and enjoyable by being vibrant and colorful.

The visuals are entertaining and engaging, using different bits of your brain, and they can be exciting, they can be beautiful. They can be really, really attention grabbing. (P1)

##### Morbid curiosity

Almost every participant recognized that for some in the public audience, part of the appeal of anatomy public engagement is the allure of the macabre. Some participants saw it as a modern version of the Victorian freakshow, underlined by a similar thrill from looking at the unusual or gruesome.

The Victorians were the experts in it, where they just absolutely reveled in the unusual, in the extreme, in the unexpected natures of human form and human anatomy… we are attracted by what's different in other people, what we like, what we don't like, what repulses us. There is that element as well of being slightly scared by anatomy. (P3)

P2 ascribed the morbid curiosity surrounding anatomy to limited exposure to death in the present day, prompting audiences to seek experiences that familiarize them with mortality.

People are just fascinated by gruesome things… on the whole, people just aren't as used to dealing with dead people. You go back to Victorian times and if your relative died in your house then you are the person who prepared them… We've outsourced it to undertakers and so people don't have that exposure anymore. (P2)

A resounding message arising from all participants was that although audiences may want, or even expect, some degree of gore from anatomy, it is incumbent on the anatomist not to engage with this. This responsibility is explored further when discussing the importance of the anatomical context.

##### Humor

All participants cited humor as a form of entertainment, noting it personalizes presentations and showcases the educator's individuality.

The use of humor, which I suppose is about being personable. That you're not just a conduit for the subject, you are allowing some of your own personality to come through. You're engaging with other humans. (P1)

Using jokes was recognized as a good way of warming an audience up at the beginning of a presentation and keeping them engaged throughout.

I usually start whatever I'm doing with a little bit of fun… because I want people to laugh, and that way they dissipate the tension… if people then start to laugh, you can feel them settle and relax. And then once you do that, you can then go into the things that are challenging, and you'll feel the temperature in the room change, and when that changes, you know you've got them. (P3)

##### Enthusiastic delivery

Most participants suggested that without a lively and enthusiastic presenter, the audience would not be entertained. If teaching is viewed as performance, effective delivery and presentation skills are crucial for engaging the audience. Much of the discussion on presentation style focused on the presenter's enthusiasm and passion for the subject.

If people wanted to know only about the work that you do, they could read your freaking paper. But when they come to hear you talk, they want to see you, and so your job is to deliver why this is important to you, and also should be important to them. (P6)

Several participants were keen to point out that presenters can lean too heavily into performance in public engagement. They believed there was a balance to be struck between presenters being passionate enough that their enthusiasm infects the audience, and going too far into showmanship and centering themselves instead of the content.

The showmanship that [Gunther von Hagens] would put on in many ways overshadowed the anatomy, and for me went too far. (P3)

Awareness of the balance between showmanship and responsibility unified participants who disliked (P3) and embraced (P1) the idea of public engagement as entertainment.

I really found Gunther Von Hagens' broadcast quite distasteful… there was a theatricality to it, and there was a brandishing of body parts which… I found quite disrespectful and distasteful. (P1)

##### Personal relevance

Throughout the interviews, a recurring idea was that “everybody has a body,” making anatomy relatable on a personal level. Nearly, all participants observed that the public has a vested interest in anatomy because people are curious about their own bodies. This personal relevance is subjective and varies, with participants describing two types of interest: a vague, general curiosity about anatomy and a more specific, individual connection.

Because your anatomy is something that everybody owns, everybody's got it… you are your own experimental Petri dish. And as a result of that, anatomy… is something that we're very happy to talk about. (P3)

Others mentioned audience members with a deeper vested interest, such as those with clinical conditions related to specific anatomical structures.

The podcast is for people who've been diagnosed with something, like ‘what do I need to understand?’ (P4)

##### Lightbulb moments

Two participants mentioned the significance of curiosity in enjoyment. They observed that invested audiences often have specific questions, whereas general audiences are typically curious about vaguely familiar topics. These participants noted the entertainment found in the process of posing a question, acquiring the necessary information, and experiencing the “lightbulb moment” when they arrive at the answer.

The minute you've got a question, you want to find the answer out, and in finding the answer out, you've done the research that gives you the sense of satisfaction. And that not knowing and then getting a result hits a button in your brain that says, ‘ooh, that was fun, I enjoyed doing that…’ (P3)

##### Gamification

Two participants discussed how anatomy readily fits into game formats which are interactive and thus engaging, while being fun and diverting. Games were seen as particularly effective in entertaining lay audience members who lack personal investment.

I think the thing that was the most successful is we used Kahoot. We did a pub quiz of anatomy… using gaming approaches to get people inquisitive. The open day is like ‘you've got to get people who came here because their kids are interested in the uni, interested in anatomy.’ (P4)

The differential effects of different types of entertainment will be touched on in the next theme.

#### Theme 3—Impact of entertainment

The perceived impacts of these forms of entertainment on a public audience broadly fall into two main categories: their impact on learning and their impact on engagement.

##### Impact on learning

Attitudes toward entertainment's impact on learning were mixed. Most participants agreed that entertainment can be beneficial for learning. It was broadly agreed that audiences learn better when they enjoy themselves. Entertainment also produces memorable experiences to which learning becomes attached.

If they can entertain as well as teach, then it probably leads to a memorable learning experience… so actually it's more likely that people will remember that session. (P5)

There's that emotional learning landmark created. You remember doing something different… and it helps you subsequently recall the information better. (P7)

Entertaining experiences were not unanimously praised. Some participants noted that forms of entertainment can be distracting and detrimental to an audience's learning.

We had a lecturer who had a story for almost every part of the body. And these stories were imparted whilst you were standing around dissecting or looking at a prosection… you can't remember what you were dissecting, but you can remember that story. (P2)

Different types of entertainment were noted to have different impacts. Fun facts, as discussed above, were sometimes described as detrimental to the recall of information. Narratives, on the other hand, were believed to be not only entertaining but also strongly tied to improved learning as they structure information into a recognizable framework that aids processing and recall.

One of the ways that storytelling helps with learning is the fact that, because we're familiar with story arcs, it helps us organize and scaffold information. (P6)

##### Impact on engagement

When considering the impact of entertainment on engagement, there were three types of engagement identified: *initial engagement*, *engagement throughout*, and *ongoing engagement*.

*Initial engagement* was seen as the audience's desire to attend a public engagement event in the first place. Participants noted that entertainment is crucial for attracting audiences, as, unlike academic settings requiring exams, there is little motivation to attend without the promise of an entertaining experience. “*Why would people come if they weren't entertained by it?*” (P2).

Educators understood the necessity of maintaining *engagement throughout* a session to hold the audience's attention. They considered entertainment as a major way to prevent attention from waning.

You've got to keep them in. If they switch off, they've all got their mobile phones and they start surfing the Internet, you've got to hook them in, haven't you? (P7)

Finally, many participants hoped that an entertaining public engagement session would inspire *ongoing engagement* with a topic, sparking an interest in the audience to go away and learn more. Many anatomists believed enjoyable events could lead to further engagement with anatomy. “*I suppose my ultimate outcome would be that people would then be wanting to learn more*.” (P1).

In terms of the differential effects of different types of entertainment, morbid curiosity and personal relevance were seen as significant drivers for initial engagement. Humor, presentation style, and narratives were noted to support engagement throughout by maintaining audience attention, while lightbulb moments could foster ongoing engagement.

##### Theme 4—Context dependency

All participants discussed this final theme that entertainment elements are subject to the context in which they are situated. The three subthemes are: (1) variation according to context: the types and impacts of entertainment vary depending on context; (2) co‐creation with audiences: audiences are involved in the creation of entertainment; and (3) anatomical context: the use of entertainment must be considered in the specific context of anatomy.

##### Variation according to context

It would be reductive to suggest that educators adjust their use of entertainment strictly based on whether they are lecturing or engaging with the public. Most participants mentioned that they tailor their entertainment methods to the setting, audience demographics, and objectives, as opposed to simply whether they were in public or not.

Your audience is going to vary depending on what kind of engagement you're doing. If you've got a pub audience, that's very different than doing a presentation in a museum, or going to an elementary school. You have to know who they are so that you can connect to them on their level. (P6)

Indeed, similar types of entertainment can be used in both university‐based education and public engagement, which relies on the context of who and what is being taught, not on the settings.

In a room with consultants from A&E, GPs, dentists, plus some intercalating medical students, that's a very different kind of audience, that's a kind of poised teaching, very much asserting yourself as the expert. Whereas if I'm doing [art‐based anatomy] with medical students, it's a bit more fun… You can up the energy, perhaps, and make it a little more informal. And I think that probably is the same with public engagement. I think it depends on my audience of teaching, more so than whether it's public engagement or not. (P7)

Together, these two quotes suggest a variety of considerations for educators when targeting their level of entertainment: different types of events delivering different content will attract different audiences. P4 gave an example of how this targeting may occur. In the context of open days, audiences may have no vested interest in the subject. Therefore, this participant described using more entertainment to maintain audience engagement.

We have an open day… it's an opportunity for future students to decide which university to attend… the open day is literally like ‘you've got to get people who came here because their kids are interested in the uni interested in anatomy’. (P4)

##### Co‐creation with audience

Audiences not only receive entertainment tailored to them, but they also contribute to its creation. The four participants most involved in public engagement (P1, P3, P4, and P6) noted how they modulated their use of entertainment in response to audience feedback. This feedback often helps refine the entertainment delivered to them.

There's an interesting iterative process with it. For instance… some of the humor you can write… [but] often you find it when you're doing it… You'll notice the bits which people are enjoying. And so the next time you do it, you'll have more of those enjoyable bits. (P1)

The availability of this feedback and modulation depends on the context. Two participants (P1 and P3) noted that a live audience allows for active engagement; in contrast presenting for television loses this co‐creation aspect of developing entertainment.

For me, engagement is a two‐way conversation. It's not a one‐way teaching. It's a two‐way reciprocal relationship… Now it might be that you're just looking down the camera, and that can be quite challenging as you have to imagine that there's somebody at the other end of it, or you're in a room filled with 500 people and you can feel the response. They are two very different things, something that is remote and something that is very personal. (P3)

##### Anatomical context

Almost every participant raised the idea that they have a responsibility not to engage with some forms of entertainment (e.g., making distasteful jokes or encouraging morbid curiosity) because their public engagement is situated within the context of anatomy.

For some, the main concern was that although human donor material is seldom used in public engagement, the field of anatomy is built upon the use of human tissue, and so the same standards must be upheld. Anatomists' knowledge comes from human donor anatomy, imposing a responsibility to treat this knowledge with respect. As such, this brought up ethical questions about the appropriateness of being entertaining and what types of entertainment are acceptable in anatomy public engagement.

These were people's fathers, mothers, grannies, grandads, who had chosen to sign a piece of paper to ensure that their remains ended up in that dissecting room and the only thing that was expected of me was that I would learn; not to be entertained by them, but to learn from them. (P3)

If you are dealing with human tissue, you have a responsibility to deal with that tissue respectfully… so you have a responsibility to these people that have donated their bodies and their families. You have a responsibility to future potential donors, you don't want to be turning off people from donating their bodies to medical science… You have a responsibility to prospective students who you don't want them to think ‘I can't possibly be a doctor because I'm turned off by this.’ There's all these layers and layers and layers of responsibility there. (P1)

For others, the main concern was of the public's perceptions of anatomy. Anatomy's troubled history casts long shadows, for both anatomists and the public. Some participants believed that the public still see anatomy as being tied to its sordid past and anticipate some degree of sensationalism from engagement. They believed that anatomy public educators have the responsibility to be aware of this history and avoid using it as entertainment and, if possible, to try to overturn this perception.

I try to make sure that I drive home the governance side of things. So ‘we're regulated by the Human Tissue Act, we get inspected.’ Because I think people still have this— ‘is it true that medical students chop hands off and put them in the toilet?’ I'm like ‘they did, years ago. Now, let me assure you, that would absolutely not happen.’… I think, there's all the historic wrongs like Alder Hey and the Redfern Inquiry. Whenever you talk about anatomy, I think you have to be really cognizant of all the grim history. (P7)

## DISCUSSION

There were three major findings from this study. The first was that the *relationship between entertainment and education is not uniformly understood or conceptualized*. The second is the *differential impacts of different entertainment types*—different types of entertainment do not all uniformly produce the same impacts. The final finding was that all aspects of this study are *situated in the anatomical context*.

### Lack of clear definition and the relationship between entertainment and education

There was little clarity among participants about the nature of the relationship between entertainment and education, which stemmed from having no clear agreement on the definition of “entertainment.” Indeed, given the subjective nature of the experience of entertainment, this was an expected finding. In this section, the variability between participants of what “entertainment” means is discussed, followed by an examination of its relationship to education, and finally an exploration of the concept of entertainment being intrinsic or extrinsic to the education content itself.

Psychological entertainment theory defines entertainment as the experience of the audience^47^; however, most participants focused on the educator's actions and whether they were “entertaining.” This may be due to the study's objective of exploring educators' attitudes toward entertainment, emphasizing their actions over audience experiences. When considering “entertainment” as an audience experience, entertainment theory distinguishes between two affective responses to stimuli: hedonic entertainment, which involves enjoyment and pleasure from humor or gameplay, and eudaimonic entertainment, which offers meaningful experiences that may not be enjoyable per se but lead to personal growth. Eudaimonic entertainment is marked by appreciation and gratification.^48^ In this study, most participants equated “entertainment” with “enjoyment,” but with a specific focus on the “fun” hedonic aspects while neglecting eudaimonic entertainment. Only one participant mentioned entertainment without enjoyment. This conflation of entertainment with fun can easily be seen to trivialize anatomy and likely contributes to the discomfort some anatomists have with using the term “entertainment,” as expressed by participants and avoidance within the wider anatomical literature.^12^, ^29^ Interestingly, some participants were more inclined to describe their teaching as “fun” or “entertaining” rather than labeling public engagement as “entertainment,” though these elements aligned with entertainment definitions as discussed by other participants and in the literature. Despite disagreement over whether the term “entertainment” was appropriate, participating anatomists were unified on which entertaining elements are appropriate for public engagement—a unity not fully reflected within the wider anatomy community, as described earlier and reported by Gomez et al.^49^

Participants' views on the relationship between entertainment and education are supported by literature, from the very nature of Entertainment‐Education^10^, ^11^ to discussions on integrating entertainment into formal education settings.^2^, ^3^ While clarification of the nature of this relationship remains elusive there are hints about how it may be considered. Taylor^12^ recognized the importance of finding an audience‐specific balance between fun elements and educational content, implying their complementarity. Francis^4^ argued that high levels of fun can effectively convey advanced content, suggesting entertainment and education can coexist without compromising learning. This is reflected in Figure 2 and P6's comment that “*I have a lot of learning outcomes to achieve, and the most effective way I have found to achieve those is actually being more engaging*.” Anatomists who conduct public engagement should position their activities somewhere in the upper right quadrant (both educational and entertaining, Figure 2), guided by the following discussion points.

A novel idea communicated in this study is distinguishing between intrinsic entertainment, such as humor relevant to the anatomical content, and extrinsic entertainment (irrelevant humor). Literature suggests that content‐related humor aids learning, whereas unrelated humor distracts.^4^ This positions entertainment relative to the content at hand, arising from or being extraneous to it. It is aligned with participants' understanding of entertainment being something they do (i.e., their communication style), emphasizing how entertainment can facilitate or hinder educational content delivery, which is a significant consideration in the next major finding.

### Differential impacts of different entertainment types

Participants identified varied types of entertainment present in anatomy public engagement, and the impacts they can have on audience learning and engagement. This section explores how perceived impacts differ by entertainment type and whether the entertaining factor is positioned intrinsically or extrinsically to the educational content.

#### Impact of narrative

Narratives are fundamental to science communication,^50^ as stories help humans comprehend and retain facts.^51^ Integrating information into narratives presents content as intrinsically entertaining and has been shown to enhance recall compared to non‐narrative delivery of information.^52^ Almost all participants discussed using narratives in alignment with broader science communication literature.^53^, ^54^ Narratives enhance learning, indicating that the main impact of narrative entertainment on audiences is its educational benefit.

#### Impact of personal relevance

The concepts underpinning personal relevance are similar to those underlying general interest formation, which can be categorized into personal and situational interest.^55^ Personal interest is an intrinsic motivation to understand a topic, often stemming from preexisting knowledge that incites curiosity. Situational interest, however, is temporary and context‐dependent, triggered by environmental stimuli such as a startling headline or a visit to a historical site. Situational interest may, therefore, be triggered by relevance to an individual, such as an interest in limb anatomy following a sporting injury, and can evolve into personal interest if sustained.^56^

Interest formation parallels anatomists' discussions on the impact of personal relevance. The promise of answering everyday questions about the human body was believed to promote situational interest in lay audiences, contributing to initial engagement which may go on to become personal interest. The interest stems from audiences having bodies of their own, situating the entertainment as intrinsic to the content, and highlighting this type of entertainment as one that centers engagement with the topic.

#### Impact of lightbulb moments

Lightbulb moments closely align with the theory of eudaimonic entertainment, specifically the enjoyment of solving puzzles and challenges.^57^ Participants described learners experiencing lightbulb moments, involving: uncovering a problem, gathering necessary information, and experiencing an epiphany of understanding. This type of entertainment is perhaps the one most intrinsically aligned with the anatomical content. It was noted that lightbulb moments foster the development of personal interest and ongoing engagement in a topic, as well as producing memorable learning landmarks which can be beneficial to learning,^58^ exemplifying the two different impacts of this entertainment type. Despite recognizing this, they did not overtly discuss how lightbulb moments may be deliberately targeted for public audiences. Encouraging such moments might involve posing a question to spark curiosity and providing structured information to solve it.

#### Impact of gamification

Educational games are well‐supported in literature, featuring diverse types like board games, escape rooms, and quizzes.^7^, ^9^, ^59^ Gamification in education offers numerous benefits, including increased audience attention and engagement throughout,^6^, ^8^ and this impact within anatomy public engagement is beginning to be explored.^60^ Evidence indicates that games can also enhance knowledge retention more effectively than standard revision methods.^6^, ^9^ Participants in this study highlighted the ability of games to extrinsically engage and hold the attention of a public audience. However, literature demonstrates the efficacy of games as a tool for learning, suggesting their usefulness extends beyond mere engagement and has a clear place within anatomical public engagement.^39^

#### Impact of morbid curiosity

The appeal of gruesome elements underlies the success of horror films^61^ and dark tourism.^62^ Similar to P2's theory, this interest stems from limited exposure to death, prompting audiences to seek mortality‐related experiences.^62^ This contributes to Body Worlds' popularity,^22^, ^62^ for which three‐quarters of visitors cited curiosity as their reason for going.^63^ Participants unanimously expressed their distaste for engaging with morbid curiosity, fearing it trivializes anatomy, impedes learning, and disrespects body donors. They noted that while it can spark initial engagement,^25^ anatomists face a challenge to ensure that voyeuristic elements do not detract from engagement with valuable learning opportunities that follow. Entertainment derived from morbid curiosity is extrinsic to anatomical content, however, it is intrinsically aligned with the public perceptions of the discipline of anatomy and its history; this forms the basis of the final finding.

### Situated in anatomical context

Much of the discussion so far could be applied to the use of entertainment in formal education settings or public engagement more broadly. The third major finding of this research highlights that entertainment in anatomy public engagement must be interpreted through the lens of anatomy. Pervading all interviews and present throughout much of the literature was the idea that situating public engagement within the anatomical context carries historical and ethical baggage, notably a perceived conflict between entertainment and dignity in a discipline reliant on human body donors.^22^, ^28^

The anatomical context is foundational to two of the discussed entertainment types. First, it encourages audiences to explore their bodies, enhancing personal relevance. On the other hand, anatomy's macabre history also attracts morbid curiosity and dark humor.^64^ As discussed in the previous section, participants felt a clear responsibility to avoid entertainment grounded in morbid curiosity. This same responsibility appears to underlie some participants' aforementioned discomfort toward describing anatomy public engagement as being “entertainment” at all, as they believed that being entertained by anatomy would inherently be disrespectful to body donors. This feeds into the misconception that entertainment is inherently hedonistic. However, the earlier findings make it clear that public engagement with anatomy (and science more generally) is often a eudaimonic entertainment experience for audiences—the entertainment comes from the gratification of learning itself. Indeed, while anatomists are not able to control why a public audience first engages with them, audience‐initiated engagement presents an opportunity to then change the perception of the discipline and emphasize respectful conversation around the human origins of anatomical information.

### Limitations

A key limitation of the study is the lack of diversity among participants, as both the lead researcher and participants were white and able‐bodied. Two of the female participants mentioned additional challenges faced by women in public engagement, particularly in finding a balance between entertainment and professionalism; being too entertaining can be perceived as being unprofessional, and not entertaining enough is seen as too serious. Although this study did not explore this issue further in the interviews, it raises questions about how the results might differ had it included participants with disabilities or from different ethnicities and backgrounds. Related to this is the limited information able to be provided about each participant's background. Further information about their outreach activities relative to their lived experiences would have provided more context to their contributions. However, given the public nature of their work, it was not possible to provide this information and ensure anonymity was maintained.

### Future research directions

This study delves into educators' attitudes toward incorporating entertainment in anatomy public engagement. However, by focusing solely on educators' perspectives, it provides only a partial view. Several participants acknowledged the importance of targeting specific audience experiences, but without understanding the audiences' views, an element of guesswork remains. To address this, future research must consider the audience experience. This can be achieved through various methods, such as qualitative interviews with diverse audience members to explore their perceptions of entertainment's impact, or observational studies of public engagement events to compare audience perceptions with observed outcomes.

Other ideas emerging from this research also warrant further exploration. The distinction between intrinsic entertainment (emerging from educational content) and extrinsic entertainment (added on top of content) is novel and requires more exploration. Investigating how different types of entertainment fit into these categories in various contexts and their differing impacts is an interesting avenue for further research. Additionally, examining the differential impacts of various entertainment styles in greater depth would provide a detailed understanding of which types most benefit learning and engagement, thus providing a rigorous framework for designing future public engagement initiatives.

The limitations mentioned highlight the complexities surrounding the use of entertainment for educators from diverse social backgrounds. While this study identified difficulties faced by educators on the basis of their gender, there is clearly much more to explore. In 2020, Professor Chris Jackson reported racial abuse for being the first black person to host a Royal Institution Christmas Lecture,^65^ indicating that such prejudices extend beyond gender and warrant further exploration.

## CONCLUSION

This research revealed a lack of consensus on defining entertainment in the context of education, with some participants unwilling to describe anatomy public engagement as “entertainment.” While the broader relationship between entertainment and education was ill‐defined, this study also identified that educators employ various entertainment forms, such as narratives, humor, games, and curiosity about one's own body, in public engagement. These forms are intrinsic to (arising from) or extrinsic (added to) the educational content and can have a variety of impacts on the audience, including impacts on engagement and learning. Notably, these are not seen as independent concepts, but rather as intricately interwoven: different types of entertainment can have different impacts on different audiences, and desired impacts can be consciously targeted by educators.

The anatomical context brings with it some unique types of entertainment, but also concerns that limit their use. This was due to the ethical and historical considerations related to the field's reliance on human body donors, and the perception that entertainment is inherently hedonistic and thus disrespectful. However, it is important to also consider that a focus on eudaimonic entertainment (related with feelings of gratification and appreciation for learning) has the potential to improve the public's awareness of ethical practices in modern anatomy and enhance audience learning.

A key point to take away from this research is the need to focus on the audience. Participants' definitions of entertainment emphasized the educator's role over the audience experience; however, many discussed how the types and impacts of entertainment can be varied to audience type. Future public engagement could benefit from a stronger focus on the type of audience they are and how to effectively use entertainment to reach them. Given that different types of entertainment have varying impacts on the audience's learning and engagement, it may be beneficial to tailor entertainment types to help meet the aims of the session. For example, incorporating games midway can maintain attention, while structuring the session around a narrative can enhance knowledge retention. Many of these findings are applicable to public engagement in various disciplines and university education more broadly. The key factor appears to be the audience and how they can be targeted, irrespective of whether the session is public‐oriented or university‐based. Thus, the lessons learned can be translated across different formats.

Ultimately, the subjective nature of entertainment hinders the identification of a single unifying definition for the relationship between entertainment and education in anatomy public engagement. The malleability of the relationship permits variability in anatomists' use of entertainment, which changes according to the desired impact and the context within which it is placed. Further research may clarify the nature of intrinsic and extrinsic entertainment or elaborate on the differential impacts of entertainment types. The relationship's complexity and variability, however, make it unlikely to fit into a simple conceptual framework. Anyone hoping for a simple instructional guide telling them how to use entertainment in their anatomy public engagement will likely be waiting a while yet. Nonetheless, anatomists looking to support the learning and engagement of public audiences must recognize that “entertainment” will be a core component of their success—even if they do not want to call it that.

## AUTHOR CONTRIBUTIONS

**Lucas D. Wilmshurst:** Conceptualization; data curation; formal analysis; investigation; methodology; writing – original draft; writing – review and editing. **Lauren Clunie:** Methodology; project administration; supervision; writing – review and editing. **Kieron Brand:** Formal analysis; writing – review and editing. **Chandini Parsan Chand:** Formal analysis; writing – review and editing. **Kat A. Sanders:** Conceptualization; methodology; project administration; supervision; writing – original draft; writing – review and editing.

## APPENDIX A SEMI‐STRUCTURED INTERVIEW PROTOCOL

### A.1

Intro—verbal re‐consent, interview recorded, withdraw at any time, start recording

1. How are you involved in anatomy public engagement?
2. In the context of anatomy public engagement, what does “entertainment” mean to you?
3. What do you believe to be the impact of such entertainment in public settings?
  - Are there settings in which it is more or less appropriate to be entertaining?
  - What forms can entertainment take in these settings?
4. Do you approach public engagement with any desired learning outcomes, and what form might they take?
  - How is this different from your planning for formal lectures?
  - Are entertaining elements a conscious part of your plan?
  - Does your use of entertainment contribute to achieving these outcomes and, if so, how?
5. Do you have a different educator personality in different settings, for example different types of public engagement and formal education?
  - Is your use of entertainment different in public and lecture settings and, if so, how?
  - Is your use of entertainment different in different public engagement settings?
6. Do you consider your public engagement to be primarily educational or primarily entertaining, and why do you think this?
  - If seen as a spectrum, how far along would you consider yourself to be?
7. To summarize, how would you define “education” and “entertainment,” and the relationship between them?
